# Mentoring New Research Center Directors Through Times of Uncertainty: The LEAD Program

**DOI:** 10.1093/geroni/igaf122.612

**Published:** 2025-12-31

**Authors:** Juanita-Dawne Bacsu, Harleah Buck

**Affiliations:** Thompson Rivers University, Kamloops, British Columbia, Canada; University of Iowa, Iowa City, Iowa, United States

## Abstract

Mentorship is vital for new research center directors to help them build leadership skills, foster diversity, and lead successful teams, especially through times of uncertainty. However, there is limited discussion on mentorship programs to support new center directors in the field of gerontology. Collaborating with the Gerontological Society of America, we developed the Leadership Excellence and Advancement of Directors (LEAD) Program as a model to provide mentorship, professional growth, and peer support for center directors. This presentation aims to: 1) outline evolving mentorship strategies to empower new centre directors in navigating unpredictable challenges and uncertainty; and ii) identify the LEAD Program as an innovative model to provide mentorship to support new directors. The LEAD Program is a trailblazing GSA initiative that was developed in response to the growing call for mentorship from incoming directors as previous leaders transitioned to retirement. The LEAD Program is quickly evolving into a hub of mentorship by: i) addressing collective challenges and leadership needs; ii) providing peer support to engage in collective problem-solving; iii) facilitating knowledge to enhance decision-making during times of uncertainty; and iv) networking to support capacity-building among center directors. Moving forward, our next steps are to assess the LEAD Program to enhance the mentorship model for future cohorts of center directors.

